# The Hepatic Plasma Membrane Citrate Transporter NaCT (SLC13A5) as a Molecular Target for Metformin

**DOI:** 10.1038/s41598-020-65621-w

**Published:** 2020-05-22

**Authors:** Jonathan Kopel, Kei Higuchi, Bojana Ristic, Toshihiro Sato, Sabarish Ramachandran, Vadivel Ganapathy

**Affiliations:** Department of Cell Biology and Biochemistry, Texas Tech University Health Sciences Center, Lubbock, 79430 Texas United States

**Keywords:** Biochemistry, Cell biology, Drug discovery

## Abstract

Metformin is the first-line treatment for type 2 diabetes. Inhibition of hepatic gluconeogenesis is the primary contributor to its anti-diabetic effect. Metformin inhibits complex I and α-glycerophosphate shuttle, and the resultant increase in cytoplasmic NADH/NAD^+^ ratio diverts glucose precursors away from gluconeogenesis. These actions depend on metformin-mediated activation of AMP kinase (AMPK). Here we report on a hitherto unknown mechanism. Metformin inhibits the expression of the plasma membrane citrate transporter NaCT in HepG2 cells and decreases cellular levels of citrate. 5-Aminoimidazole-4-carboxamide ribonucleotide (AICAR), an AMPK activator, elicits a similar effect. The process involves a decrease in maximal velocity with no change in substrate affinity. The decrease in NaCT expression is associated with decreased mRNA levels. AMPK inhibits mTOR, and the mTOR inhibitor rapamycin also decreases NaCT expression. The transcription factor downstream of AMPK that is relevant to cAMP signaling is CREB; decreased levels of phospho-CREB seem to mediate the observed effects of metformin on NaCT. Citrate is known to suppress glycolysis by inhibiting phosphofructokinase-1 and activate gluconeogenesis by stimulating fructose-1,6-bisphophatase; therefore, the decrease in cellular levels of citrate would stimulate glycolysis and inhibit gluconeogenesis. These studies uncover a novel mechanism for the anti-diabetic actions of metformin.

## Introduction

Diabetes remains an increasing healthcare burden as more patients with chronic and poorly controlled diabetes develop several cardiovascular and metabolic abnormalities^1–3^. Clinicians manage the symptoms and progression of diabetes through several pharmacological agents that decrease hepatic gluconeogenesis, increase glucose disposal, increase insulin sensitivity, or increase insulin secretion. Currently, metformin (1,1-dimethylbiguanide) remains the first-line treatment for type 2 diabetic patients and is routinely combined with other glucose-lowering agents for optimal glucose control^4,5^. Several clinical trials have shown metformin lowers the risk of myocardial infarction and mortality and improves weight loss among type 2 diabetic patients^6,7^. Furthermore, metformin’s only known adverse side effect is lactic acidosis, which occurs at large doses. With its strong safety profile and clinical benefit, metformin is routinely given to patients with hepatic impairment, heart failure, and chronic kidney disease, who have limited therapeutic options for type 2 diabetes management^4^. In recent years, metformin has gathered interest from the academic and medical communities for its potential applications in cancer prevention and anti-aging properties^8–11^.

Despite its widespread use, the pharmacological mechanism of metformin remains poorly understood and is an area of active investigation. Especially now that metformin has therapeutic uses in multiple diseases in addition to diabetes, a better understanding of the mechanisms of action is warranted for this drug. What is currently known on metformin’s mechanism of action in the treatment of type 2 diabetes include the following^12–18^: (i) inhibition of complex I in electron transport chain, leading to an increase in AMP/ATP ratio and NADH/NAD^+^ ratio, (ii) inhibition of AMP deaminase, which also causes an increase in AMP/ATP ratio, (iii) inhibition of mitochondrial α-glycerophosphate dehydrogenase, consequently suppressing α-glycerophosphate shuttle and increasing cytoplasmic NADH/NAD^+^ ratio, and (iv) direct activation of AMP kinase (AMPK). As such, metformin has multiple targets, but all these effects collectively lead to an increase in NADH/NAD^+^ ratio and AMPK activity, which together suppress gluconeogenesis in liver. Extrahepatic actions of metformin that might also be relevant to its anti-diabetic activity is the alteration in gut microbiome and the resultant impact on enteroendocrine cells that regulate insulin secretion by pancreatic islets via the gut-pancreas axis^19–21^. Here we report on yet another pharmacologic target for metformin, namely the plasma membrane Na^+^-coupled citrate transporter (NaCT/SLC13A5) whose expression in liver is suppressed in response to metformin. The identification of this novel target sheds new light on the complex mechanisms involved in the anti-diabetic effects of this drug.

## Results

### Inhibition of NaCT-mediated citrate uptake in HepG2 cells by metformin and AICAR

**Figure 1:**
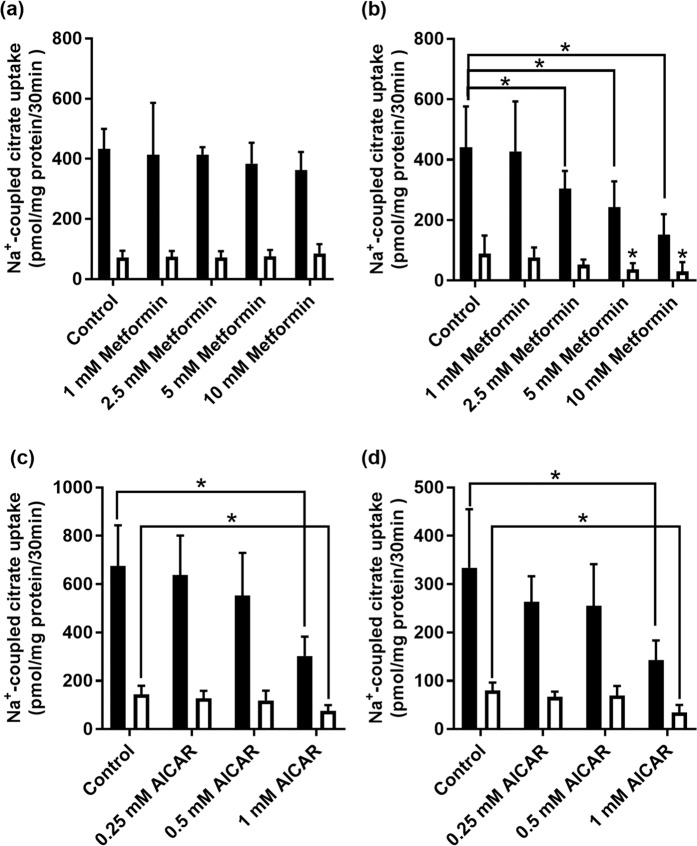
Effect of metformin treatment on Na^+^-coupled citrate uptake in HepG2 cells cultured under conditions of high-glucose (20 mM) (**a**) or physiologic concentration of glucose (5 mM) (**b**). HepG2 cells were treated with metformin (0–10 mM) for 24 h. Uptake of [^14^C]-citrate was measured in presence (closed bars) or absence (open bars) of 10 mM Li^+^. Each column represents the mean ± S.D. (n = 9). *P < 0.05 indicates a significant difference *vs* the corresponding control in presence or absence of Li^+^. Effect of AICAR treatment on Na^+^-coupled citrate uptake in HepG2 cells cultured under conditions of high-glucose (20 mM) (**c**) or physiologic concentration of glucose (5 mM) (**d**). HepG2 cells were treated with AICAR (0–1 mM) for 24 h. Uptake of [^14^C]-citrate was measured in presence (closed bars) or absence (open bars) of 10 mM Li^+^. Each column represents the mean ± S.D. (n = 9). *P < 0.05 indicates a significant difference *vs* the corresponding control in presence or absence of Li^+^.

As citrate is one of the most effective modulators of glucose metabolism via inhibition of glycolysis (allosteric inhibition of phosphofructokinase-1) and activation of gluconeogenesis (allosteric activation of fructose-1,6-bisphosphatase) in liver, we asked if metformin has any effect on the Na^+^-coupled citrate transporter NaCT/SLC13A5 in the human liver cell line HepG2. This transporter is known to be expressed in the sinusoidal membrane in hepatocytes suitable to mediate the uptake of citrate from blood into cells^22^. This transporter is expressed in HepG2 cells^22,23^. We examined the effects of metformin on NaCT in cells cultured under conditions of physiologic glucose levels (~5 mM) or of hyperglycemia (~20 mM). Cells were treated with various concentrations of metformin for 24 h, following which citrate uptake was measured in a NaCl-containing uptake medium. In cells cultured with 20 mM glucose, metformin did not have any noticeable effect (Fig. 1a). However, in cells cultured with 5 mM glucose, metformin at 5 mM and 10 mM had a significant (P < 0.05) inhibitory effect on citrate uptake (Fig. 1b). To confirm that the observed effect of metformin was due to NaCT-mediated citrate uptake, we monitored citrate uptake in control and metformin-treated cells in the presence of Li^+^ in the uptake buffer. Li^+^ is a specific activator of human NaCT^23,24^. Citrate uptake was stimulated by Li^+^ in HepG2 cells irrespective of glucose concentration used while culturing the cells, substantiating NaCT involvement in the citrate uptake. Metformin treatment at 2.5 mM, 5 mM, and 10 mM significantly (P < 0.05) decreased citrate uptake when measured in the presence of Li^+^; again, this effect was observed however only in cells cultured in the presence of 5 mM glucose (Fig. 1b).

As one of the pharmacologic actions of metformin is activation of AMPK, we repeated the experiments with AICAR, which is more potent than metformin as an activator of AMPK. The results with AICAR were similar to those with metformin except that the effects were more pronounced. Furthermore, we were able to detect inhibition of citrate uptake with AICAR treatment in cells cultured in the presence of high glucose (Fig. 1c) as well as physiologic levels of glucose (Fig. 1d).

**Figure 2:**
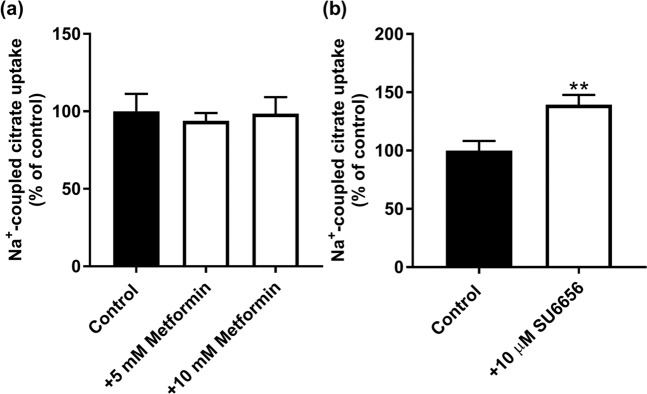
(**a**) Lack of any direct effect of metformin on Na^+^-coupled citrate uptake in HepG2 cells. Confluent cells were used for measurement of citrate uptake with and without metformin directly added to the uptake buffer (i.e., metformin was present during uptake). (**b**) Effect of SU6656, an inhibitor of AMPK, on citrate uptake in HepG2 cells. Cells were treated with and without the inhibitor (10 μM) for 24 h and then the medium was removed and the regular uptake buffer was used to monitor citrate uptake. **P < 0.01 indicates a significant difference *vs* the control.

In the afore-mentioned experiments, cells were treated with metformin and then citrate uptake was measured in the absence of metformin. To ascertain that metformin had no direct effect on citrate uptake, we monitored Na^+^-coupled citrate uptake in control cells cultured in the presence of physiologic levels of glucose; uptake was measured with and without metformin added during uptake. We found no effect (Fig. 2a). To confirm the involvement of AMPK in the observed effects of metformin and AICAR on citrate uptake, we used a pharmacologic inhibitor of the enzyme (SU6656; IC_50_, 0.22 μM). The rationale was that if metformin and AICAR suppress citrate uptake via activation of AMPK, treatment of the cells with an inhibitor of AMPK should have the opposite effect. That was indeed the case. Exposure of HepG2 cells to SU6656 (10 μM) led to a significant increase in Na^+^-coupled citrate uptake (Fig. 2b).

To corroborate the findings in HepG2 cells, we used another human liver cell line (Huh-7) that was positive for NaCT functional activity. We also examined the non-transformed human liver cell line THLE-2; this cell line did not show any detectable activity for Na^+^-coupled citrate uptake. Therefore, we tested the effects of metformin and AICAR on NaCT activity in Huh-7 cells. We found that treatment of these cells with 5 mM metformin or 2.5 mM AICAR for 24 h reduced Na^+^-coupled citrate uptake significantly (30 ± 5% for metformin; 43 ± 3% for AICAR).

### Effects of metformin and AICAR on kinetic parameters of citrate uptake

**Figure 3:**
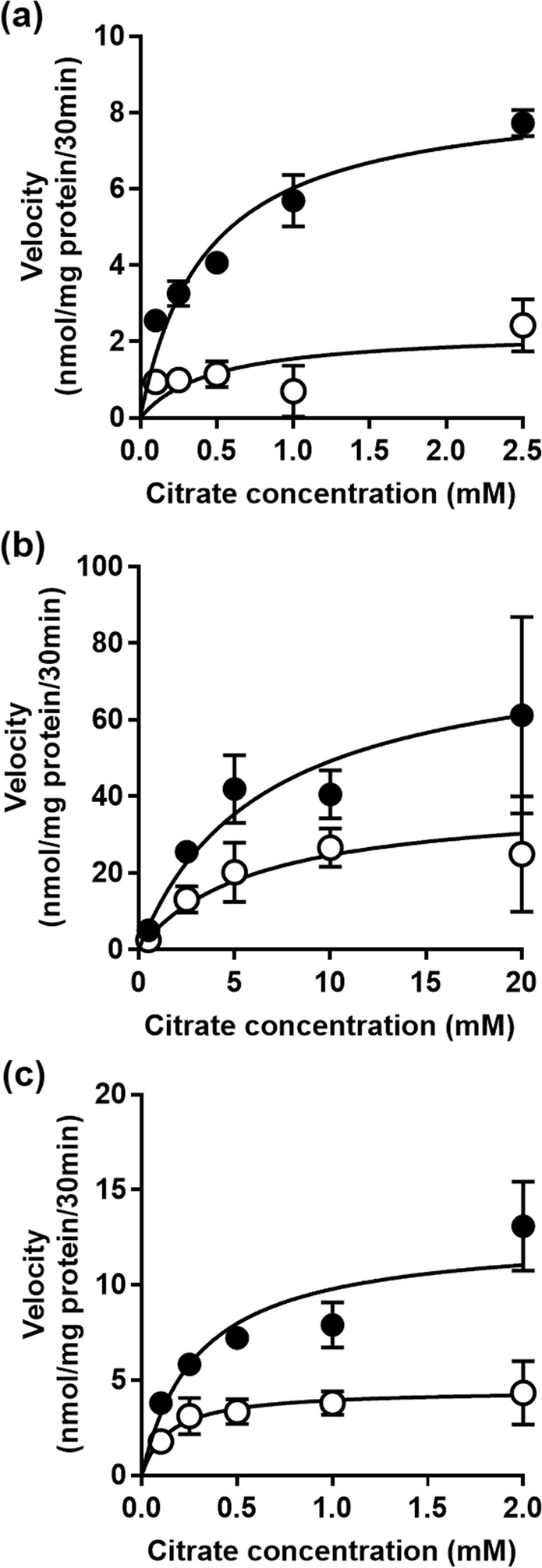
Kinetic analysis of citrate uptake in metformin- and AICAR-treated HepG2 cells. Cells were treated in the absence (control) or presence of metformin (5 mM) or AICAR (1 mM) for 24 h. For metformin, control and treatment cells were cultured only in the presence of physiologic concentration of glucose (5 mM), and uptake measurements were made only in the presence of 10 mM Li^+^ (**a**). For AICAR, control and treatment cells were cultured in the presence of 20 mM glucose, and uptake measurements were made in the absence (**b**) or presence (**c**) of 10 mM Li^+^. [^14^C]-Citrate was used as a tracer to monitor citrate uptake and unlabeled citrate was used to alter the concentrations. Each point is the mean ± S.D. of 6–9 determinations, and the data represent only the carrier-mediated uptake after correction for non-saturable component as described in the Materials and Methods section.

We then examined the effect of metformin and AICAR treatment on the kinetic parameters of NaCT-mediated citrate uptake. In the case of metformin, the experiments were performed only with cells cultured in the presence of physiologic concentration of glucose (5 mM) as there was no inhibition with cells cultured in the presence of 20 mM glucose. Furthermore, as citrate uptake was lower in cells cultured in the presence of 5 mM glucose than in cells cultured in the presence of 20 mM glucose, we used 10 mM Li^+^ for the kinetic analysis using 5 mM metformin to reduce variability and improve accuracy. The data are given in Fig. 3a. A summary of the values for the kinetic parameters maximal velocity (*V*_max_) and Michaelis-Menten constant (*K*_m_) is given in Supplementary Table 1. Citrate uptake was saturable in control and metformin-treated cells. In control cells, the values for *V*_max_ and *K*_m_ were 11.2 ± 1.4 nmol/30 min/mg protein and 0.48 ± 0.16 mM, respectively. The corresponding values for metformin-treated cells were 3.9 ± 1.4 nmol/30 min/mg protein and 0.74 ± 0.44 mM. Metformin decreased the maximal velocity of the transport system without having significant effect on substrate affinity. The experiment was repeated in the AICAR-treated cells. As AICAR treatment inhibited citrate uptake even in cells cultured in the presence of 20 mM glucose, kinetic analysis with AICAR was done with cells cultured under these conditions. The data for kinetic analysis, done in the absence (Fig. 3b) or presence (Fig. 3c) of 10 mM Li^+^, showed that AICAR also decreased the maximal velocity without having any effect on Michaelis constant. In the absence of Li^+^, the *V*_max_ and *K*_m_ values for the control cells were 80.7 ± 10.0 nmol/30 min/mg protein and 6.4 ± 1.3 mM, respectively. The corresponding values for AICAR-treated cells were 39.4 ± 6.3 nmol/30 min/mg protein and 6.0 ± 1.6 mM, respectively. Similar effects were observed for AICAR when citrate uptake was measured in the presence of Li^+^. Furthermore, the ability of Li^+^ to transform the transport process via NaCT from a low-affinity/high-capacity type to a high-affinity/low-capacity type was clearly evident in the change of *V*_max_ and *K*_m_ in the control cells. A summary of the values for the kinetic parameters maximal velocity (*V*_max_) and Michaelis-Menten constant (*K*_m_) is given in Supplementary Table 1.

### Metformin and AICAR decrease NaCT expression

**Figure 4:**
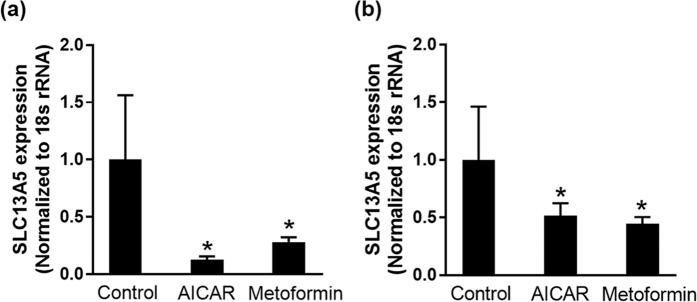
Effect of metformin and AICAR treatment on NaCT mRNA levels in HepG2 cells. HepG2 cells were treated with 1 mM AICAR or 5 mM metformin in 20 mM-glucose medium (**a**) and 5-mM glucose medium (**b**) for 24 h. Data represent mean ± S.D. from three independent experiments. *P < 0.05 indicates a significant difference *vs* the control.

The decrease in *V*_max_ with minimal change in *K*_m_ by treatment with metformin and AICAR suggest a decrease in the expression of the transporter because Michaelis-Menten constant is an inherent property of the transporter which is not influenced by the amount of the transporter protein whereas maximal velocity is directly proportional to the amount of the transporter protein. Therefore, we examined the steady-state levels of NaCT mRNA in control and treated cells by quantitative real-time PCR (Fig. 4). The mRNA levels were significantly (P < 0.05) decreased in cells treated with metformin or AICAR. Interestingly, the mRNA decrease was seen irrespective of whether the cells were cultured in the presence of 5 mM or 20 mM glucose even though the inhibitory effect with metformin was seen only in cells cultured in the presence of physiological (5 mM) glucose.

### mTOR inhibition by rapamycin mimics the effects of metformin and AICAR on NaCT expression and function

**Figure 5:**
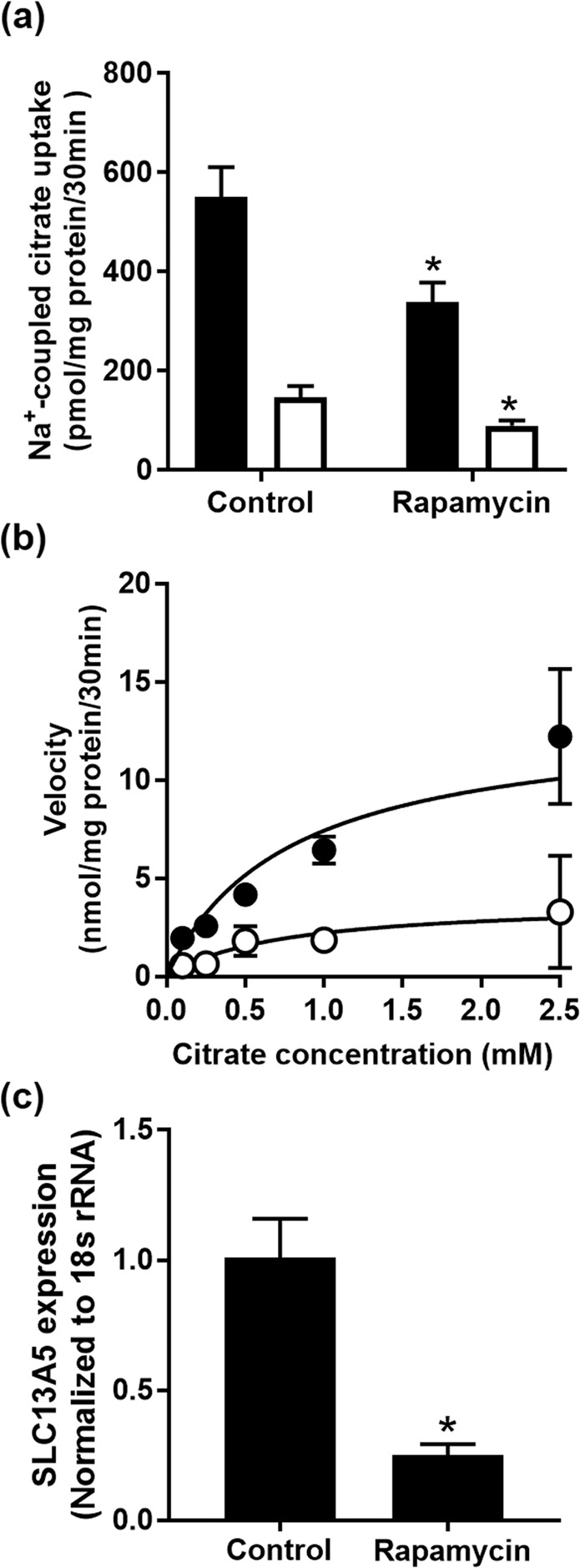
Effect of rapamycin on citrate uptake and NaCT expression in HepG2 cells. (**a**) HepG2 cells were cultured in 20-mM glucose medium and treated with 20 µM rapamycin for 24 h. Uptake of citrate was measured in the presence (closed bars) or absence (open bars) of 10 mM Li^+^. Each column represents the mean ± S.D. of six determinations. (**b**) Kinetic analysis of citrate uptake in control (closed circles) and rapamycin-treated (open circles) cells; data represent only carrier-mediated, saturable uptake, measured in the presence of 10 mM Li^+^. (**c**) Total RNA was isolated from control and rapamycin-treated cells and used for qPCR to determine NaCT mRNA levels. *P < 0.05 compared to the corresponding controls.

Activation of AMPK phosphorylates mTOR and inhibits its activation and transduction pathway involved in anabolic processes, including fatty acid synthesis and sterol synthesis^25^. Given that metformin and AICAR activate AMPK, we investigated whether mTOR influences the function and expression of NaCT. For this, we treated HepG2 cells with 20 μM rapamycin, a well-known mTOR inhibitor^26^, for 24 h in high-glucose (20 mM) medium and then monitored the expression and activity of NaCT. Treatment with rapamycin decreased citrate uptake and the inhibition of uptake was observed with or without Li^+^ (Fig. 5a). We then performed kinetic analysis to determine the impact of rapamycin on V_max_ and K_m_ for the uptake process, and the uptake measurements were done only in the presence of Li^+^. The effect of rapamycin was associated primarily with a decrease in *V*_max_ (control, 13.2 ± 2.6 nmol/30 min/mg protein; treatment, 3.9 ± 1.4 nmol/30 min/mg protein) and no change in *K*_m_ (control, 0.78 ± 0.24 mM; treatment, 0.74 ± 0.43 mM) (Fig. 5b). This decrease in citrate uptake was associated with a significant (P < 0.05) decrease in NaCT mRNA (Fig. 5c).

We also monitored the effects of co-treatment of the cells with metformin or AICAR with and without rapamycin on NaCT activity. The rationale was as follows: if metformin and AICAR suppress NaCT expression via activation of AMPK and subsequent inhibition of mTOR, additional presence of an mTOR inhibitor should not potentiate the effects of metformin or AICAR. Treatment of the cells with 5 mM metformin or 1 mM AICAR decreased Na^+^-coupled citrate uptake as observed in previous experiments; co-treatment with 10 μM rapamycin did not enhance the inhibitory effects of metformin or AICAR any further (Supplementary Fig. 1).

**Figure 6:**
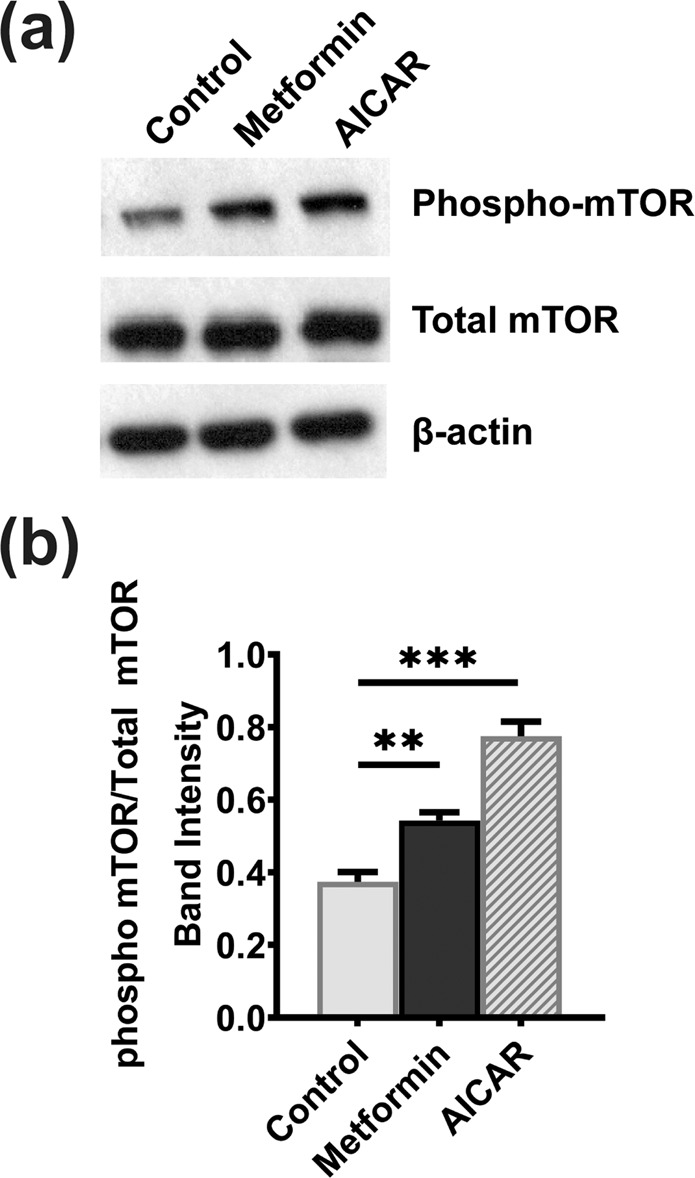
mTOR phosphorylation in HepG2 cells in response to treatment of the cells with metformin and AICAR. HepG2 cells were treated with 1 mM AICAR or 10 mM metformin for 24 h while culturing the cells in 5-mM glucose medium. (**a**) Western blot analysis depicting the phospho-mTOR (Ser-2448) in cell lysates from control and treated HepG2 cells. (**b**) Intensities of bands that correspond to the phospho-mTOR levels normalized to the respective total mTOR levels. Columns represent mean values of three independent experiments per group ± SD. **P < 0.01, ***P < 0.001, indicating a significant difference *vs* the untreated control.

To confirm directly the activation of AMPK in HepG2 cells treated with metformin or AICAR, we monitored the phosphorylation status of Ser-2448 in mTOR, which is one of the sites for AMPK-dependent phosphorylation^27^. Western blot analysis of lysates from control cells and metformin- and AICAR-treated cells showed increased phosphorylation of mTOR in response to treatment with both the compounds (Fig. 6).

### Non-involvement of SREBP-1 in metformin-mediated down-regulation of NaCT

SREBP-1 and -2 are the transcription factors that control sterol synthesis in response to AMPK-mTOR pathway^28^. As cytoplasmic citrate is the sole source of acetyl CoA for the synthesis of fatty acids and sterols, we hypothesized that SREBP-1 and -2 are involved in the suppression of NaCT expression by metformin and AICAR. We did find the presence of two putative binding sites for SREBP-1 in the promoter of human *SLC13A5* upstream of the transcription start site (Supplementary Fig. 2A). To test our hypothesis, we first determined if SREBP-1 binds to *SLC13A5* promoter using chromatin-immunoprecipitation (ChIP) assay. The ChIP primers used for the experiment are given in Supplementary Table 2. These experiments failed to detect the binding of SREBP-1 to either of the binding sites on the promoter (Supplementary Fig. 2B). We used LDL receptor as a positive control for the binding of SREBP-1 to validate our experimental conditions. ChIP assay was able to show the binding of SREBP-1 to LDL receptor promoter. To further confirm the non-involvement of this particular transcription factor in NaCT regulation, we used the pharmacologic agent fatostatin, because this compound is widely used to antagonize the biologic effects of SREBP-1 and -2 by preventing the cleavage of SREBP-1 and -2 from the Golgi apparatus^29^. If these transcription factors are involved in the regulation of NaCT expression, treatment of cells with fatostatin is expected to decrease the expression and activity of the transporter. However, contrary to our expectations, fatostatin increased the activity of NaCT as evident from the increased uptake of citrate in fatostatin-treated cells than in control cells confirming that neither SREBP-1 nor SREBP-2 is involved in the regulation of NaCT expression (Supplementary Fig. 2C).

### Involvement of CREB in metformin-mediated suppression of NaCT expression

**Figure 7:**
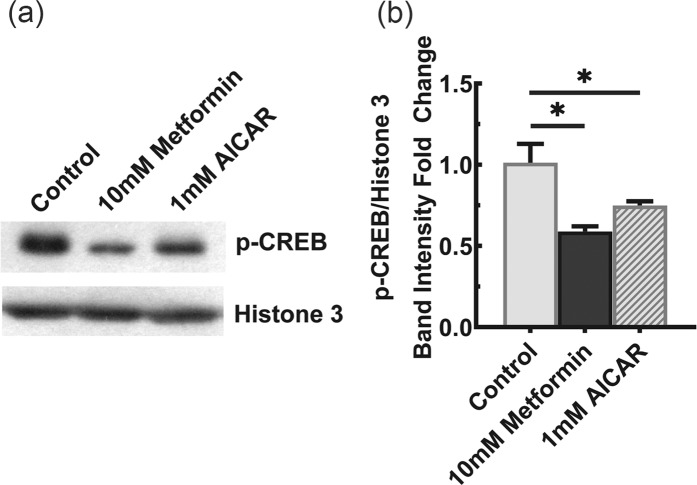
CREB phosphorylation in HepG2 cells in response to treatment of the cells with metformin and AICAR. HepG2 cells were treated with 1 mM AICAR or 10 mM metformin for 24 h while culturing the cells in 5-mM glucose medium. (**a**) Western blot analysis depicting the phospho-CREB in nuclear extracts from control and treated HepG2 cells. Full-length blots for phospho-CREB and Histone-3 are presented in Supplementary Fig. 2 (upper panel) and 3 (lower panel), respectively. (**b**) Intensities of bands that correspond to the phospho-CREB levels normalized to the respective Histone-3 levels. Columns represent mean values of three independent experiments per group ± SD. *P < 0.05 indicates a significant difference *vs* the untreated control.

Given the lack of involvement of SREBP-1 and -2 in the regulation of NaCT expression, we shifted our attention to (Cyclic Adenosine Monophosphate)-Responsive Element Binding Protein (CREBP), which is another known regulator of NaCT transcription^30^. We hypothesized that AICAR and metformin reduce the expression of NaCT by decreasing the CREBP activity. To test this hypothesis, we monitored the phosphorylation status of CREBP, a known indicator of its activity, via western blot upon metformin and AICAR treatment. The experiment was conducted in nuclear lysates collected from HepG2 cells which were treated with 10 mM metformin and 1 mM AICAR for 24 h while culturing the cells in the presence of 5 mM glucose. As Shown in Fig. 7, when compared to the untreated cells, both metformin and AICAR elicited a significant (P < 0.05) decrease in CREBP phosphorylation.

### Metformin and AICAR decrease intracellular levels of citrate

**Figure 8:**
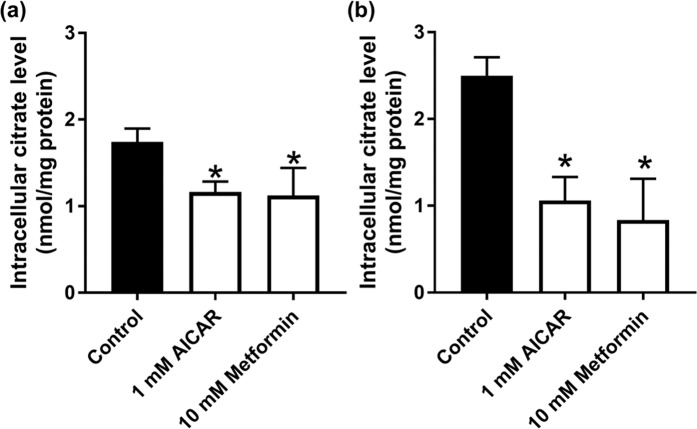
Intracellular levels of citrate in control and metformin- and AICAR-treated HepG2 cells. Cells were cultured either in 20-mM glucose medium (**a**) or 5-mM glucose medium (**b**) and treated with or without 1 mM AICAR or 10 mM metformin for 24 h. Following this treatment, cells were exposed to 200 µM citrate for 30 min in a NaCl-containing uptake buffer. Cells were then collected and used for measurement of citrate. Data represent mean ± S.D. (n = 6). *P < 0.05 indicates a significant difference *vs* the corresponding control.

NaCT is expressed exclusively in the blood-facing sinusoidal membrane of hepatocytes^22^; therefore, the physiological function of this transporter must be the uptake of citrate from blood into cells. As treatment of HepG2 cells with metformin and AICAR reduces the mRNA expression and the activity of NaCT, intracellular citrate levels are expected to decrease in cells treated with both the compounds. But the regular medium used for the culture of HepG2 cells does not contain citrate and therefore the cells are not exposed to it. To test our hypothesis that NaCT controls cellular levels of citrate, we cultured HepG2 cells in the regular medium during treatment with 10 mM metformin and 1 mM AICAR using 5 mM and 20 mM glucose culture medium for 24 h. The control, 10 mM metformin, and 1 mM AICAR treated cells were exposed to 200 µM citrate for 30 min in a NaCl-containing uptake buffer. These experiments showed that irrespective of the glucose concentration in the culture medium, metformin and AICAR significantly (P < 0.05) decreased the cellular levels of citrate compared to untreated control cells (Fig. 8). The decrease was however more pronounced in cells cultured under conditions of physiologic concentrations of glucose (60–70%) than under conditions of high glucose (30%).

## Discussion

The most critical step in glycolysis and gluconeogenesis is the conversion of fructose-6-phosphate to fructose-1,6-bisphosphate and vice versa. At this step, phosphofructokinase-1 mediates the forward reaction in glycolysis whereas fructose-1,6-bisphosphatase mediates the reverse reaction in gluconeogenesis. Citrate is a potent regulator of this step where it functions as an allosteric inhibitor of phosphofructokinase-1 and an allosteric activator for fructose-1,6-bisphosphatase. As such, cellular levels of citrate serve as an important determinant of the relative rates of glycolysis and gluconeogenesis in liver; high levels suppress glycolysis and stimulate gluconeogenesis and vice versa. The levels of citrate in the cytoplasm of liver cells are controlled by its release from mitochondrial matrix by SLC25A1, a transporter in the innermitochondrial membrane, and also by its entry from blood by SLC13A5, a transporter in the plasma membrane^31–33^. In essence, citrate inhibits glycolysis and accelerates gluconeogenesis. This is exactly the opposite of what AMPK does! As activation of AMPK is at least one of the pharmacologic actions of the anti-diabetic drug metformin, we became interested in investigating the potential impact of this drug on the plasma membrane citrate transporter as a means to control cellular levels of citrate and consequently the relative rates of glycolysis and gluconeogenesis. The reciprocal relationship between AMPK and citrate in terms of their effects on glycolysis and gluconeogenesis predicted that metformin might decrease cellular levels of citrate in connection with an increase in AMPK activity and that one of the ways the drug could accomplish this is via suppression of the expression and activity of SLC13A5. With this rationale, we undertook the present study.

The results of the studies can be summarized as follows. Treatment of the human liver cell line HepG2 with metformin decreases the expression of SLC13A5 and this effect is accompanied with a decrease in SLC13A5 mRNA. The involvement of AMPK activation in this process is evidenced with a similar effect when the cells are treated with AICAR, a positive control as an AMPK activator. In the case of both the compounds, the decrease in transport function of SLC13A5 is due to a decrease in maximal velocity with no significant change in substrate affinity, which suggests a decrease in transporter density in the plasma membrane. Unfortunately, we were unable to demonstrate the change in the levels of the transporter protein due to lack of specific antibodies. mTORC1 lies downstream of AMPK; AMPK activation, as expected from treatment with metformin or AICAR, leads to inhibition of mTORC1. Accordingly, the effects of these two drugs on SLC13A5 are reproducible with rapamycin, a known inhibitor of mTORC1. SREBP is one of the transcription factors controlled by mTORC1^28^. Since this transcription factor is involved in the regulation of sterol and fat biosynthesis^28^ and also because citrate is the most important source of carbon for sterol and fat biosynthesis, we thought that the inhibitory effects of metformin and AICAR on SLC13A5 might be mediated via this transcription factor. But the results turned out to be negative. We then turned to cAMP signaling pathway, which is known to stimulate SLC13A5 expression^30^. We found that metformin and AICAR decrease the phosphorylation of CREB. The suppression of SLC13A5 expression induced by metformin and AICAR is accompanied with a decrease the cellular levels of citrate.

**Figure 9:**
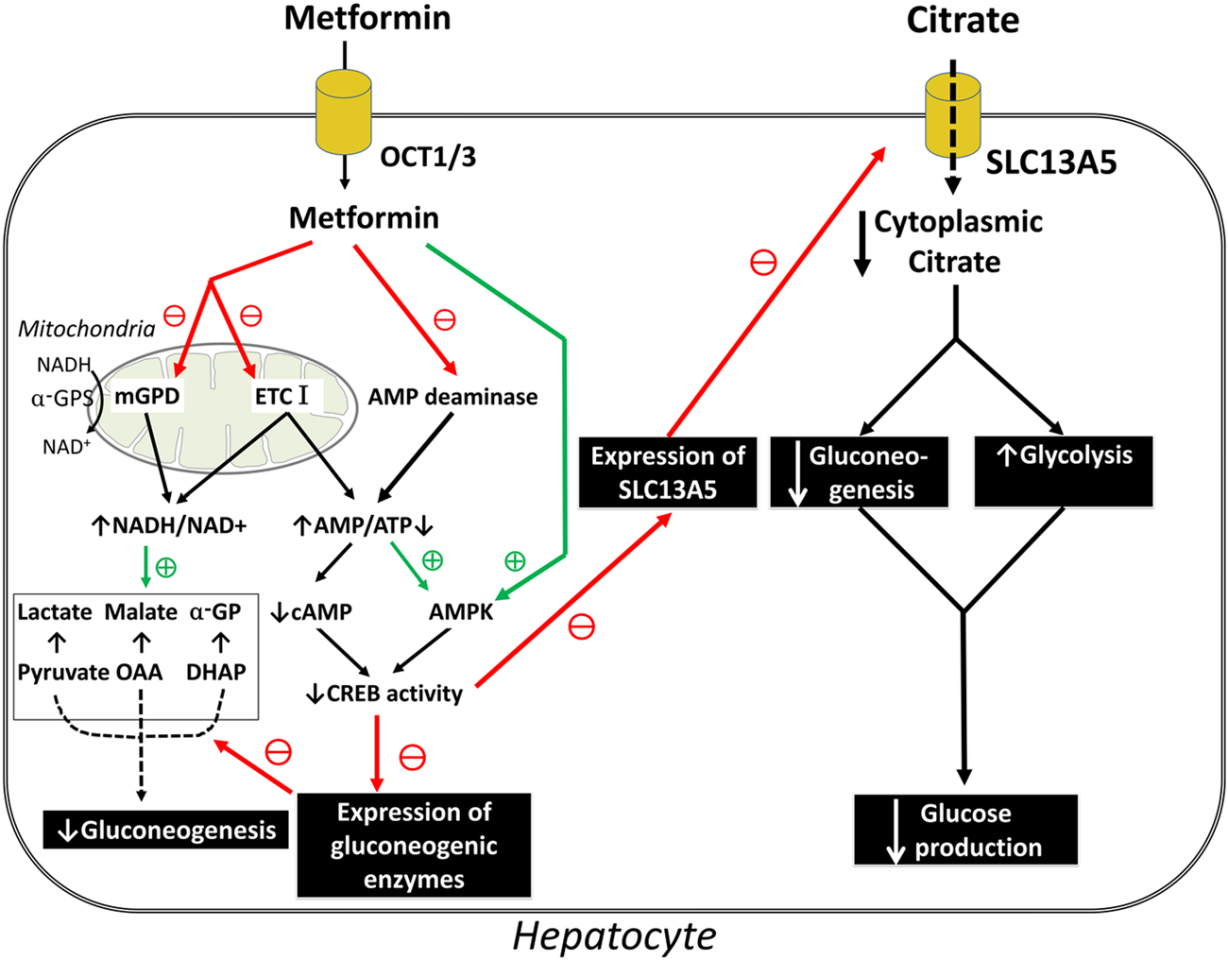
Schematic model for what is known and what is new for the mechanism of action for metformin.

These data provide a novel pharmacologic mechanism for the anti-diabetic efficacy of metformin. By suppressing SLC13A5 expression, the drug decreases citrate uptake from blood and reduces cellular levels of citrate in liver. This is expected to accelerate glycolysis and inhibit gluconeogenesis. These new findings do not dispute any of the previously described mechanisms of metformin action (Fig. 9); they simply uncover another mode of action for this drug.

In our studies, we found metformin to elicit its suppressive effects on SLC13A5 expression at low millimolar concentrations. Previous studies have shown that metformin inhibits Complex I in electron transport chain, but this inhibition is seen only at 5 mM^34,35^. Plasma concentrations of metformin are ~40 µM^34^. However, we do not know the concentrations of this drug in portal blood that carries the drug from the intestinal tract and takes it to the liver, but this concentration is expected to be many fold higher than in systemic blood. Therefore, it is possible that chronic exposure of metformin could affect the expression of SLC13A5 in hepatocytes.

While our studies have identified a novel mechanism of action for the anti-diabetic effects of metformin, which focus mostly on the liver, they raise some concerns regarding the potential influence of metformin on the brain. As loss-of-function mutations in SLC13A5 lead to severe epilepsy and encephalopathy very early in life^36^, the possible detrimental effects of chronic use of metformin on brain function need to be considered. SLC13A5 is expressed in brain^31^, mostly in neurons^37^. Therefore, the transporter is likely to play an important role in the maintenance of cytoplasmic concentrations of citrate by mediating the cellular entry of citrate from extracellular fluid. Cytoplasmic citrate is the primary carbon source for synthesis of the neurotransmitters acetylcholine, glutamate, and γ-aminobutyrate^36^. Consequently, the potential risk of affecting the brain levels of these neurotransmitters with chronic use of metformin needs to be considered. This could be relevant at least to individuals with compromised activity of the transporter as in parents of children affected by loss-of-function mutations in SLC13A5. These parents are carriers for the mutation and hence have one normal copy and one mutant copy, but with no clinical symptoms of SLC13A5 deficiency. Metformin might have an increased probability of affecting brain function in such carriers.

## Materials and Methods

### Materials

[^14^C]-Citrate (specific radioactivity, 113 mCi/mmol) was purchased from Moravek Biochemicals (Brea, CA, USA). HepG2 cells were purchased from the American Type Culture Collection (Manassas, VA, USA). 5-Aminoimidazole-4-carboxamide ribonucleotide (AICAR) and Rapamycin were purchased from Alfa Aesar (Haverhill, MA, USA). Metformin was purchased from Sigma Aldrich (St. Louis, MO, USA), and SU6656 was from Tocris (Minneapolis, MN, USA).

### Cell culture

HepG2 cells were maintained in Dulbecco’s Modified Eagle Medium (DMEM) with high or physiologic levels of glucose. High-glucose (20 mM glucose) DMEM (Corning, NY, USA) was supplemented with 10% fetal bovine serum (FBS), 1% penicillin/streptomycin, 1 mM pyruvate, and 1 mM non-essential amino acids. Physiologic-glucose (5 mM) DMEM (Sigma Aldrich, St. Louis, MO, USA) was supplemented with 2.5% FBS and 1% penicillin/streptomycin. Huh-7 and THLE-2 cells were obtained from American Tissue Culture Collection and were cultured in respective culture media recommended by the company.

### Uptake measurement

HepG2 cells were seeded at 2 ×10^5^ cells per well on 24-well culture plates and cultured in 20 mM- or 5 mM-glucose medium for 3–4 days. After 24-h treatment with AICAR, metformin, or rapamycin, the transport function of NaCT was monitored by measuring the uptake of [^14^C]-citrate using a NaCl uptake buffer (140 mM NaCl, 5.4 mM KCl, 1.8 mM CaCl_2_, 0.8 mM MgSO_4_, and 5 mM glucose, buffered with 25 mM Hepes/Tris, pH 7.5) with or without 10 mM LiCl, respectively. The uptake measurements were recorded for a 30-min period at 37 °C. The amount of uptake was corrected by protein content measured by a Pierce™ BCA Protein Assay Kit (Thermo Fisher Scientific, Waltham, MA, USA).

To estimate the kinetic parameters, the initial uptake velocity of citrate at each concentration (0.1–20 mM, for 30 min) was calculated by subtraction of non-saturable component from the total uptake velocity. Non-saturable component was estimated by subjecting the uptake data to a transport model consisting of a single saturable transport system with Michaelis-Menten saturable kinetic characteristics and a non-carrier-mediated, non-saturable, diffusional component. The initial uptake velocity for the saturable component was plotted and analyzed using the Michaelis-Menten equation by GraphPad Prism 7.01 software (Graphpad, San Diego, CA, USA).

### RNA isolation and quantitative PCR (qPCR)

Total RNA that was extracted using TRIzol reagent (Thermo Fisher Scientific, Waltham, MA, USA) and its concentration was estimated by NanoDrop Spectrophotometer 2000 (Thermo Fisher Scientific). cDNA was synthesized using High Capacity cDNA Reverse Transcription kit (Thermo Fisher Scientific). qPCR was performed to measure relative mRNA levels using SYBR Green detection system (BioRad, Hercules, CA, USA). Amplification and detection were carried out on an Applied Biosystems 7500 Real-Time PCR System (Thermo Fisher Scientific,). The relative mRNA expression was determined by the 2^−ΔΔ^Ct method. 18S was used as a housekeeping gene to normalize the relative expression level in samples. The PCR primer sequences are provided in Supplementary Table 3.

### Measurement of intracellular citrate

HepG2 cells were seeded at 2 ×10^5^ cells per well in 24-well culture plates and cultured in high-glucose medium for 3–4 days. After 24-h treatment with AICAR or metformin using a 5 mM glucose concentration, cells were exposed to physiological citrate concentration (200 µM) citrate in a NaCl uptake buffer (140 mM NaCl, 5.4 mM KCl, 1.8 mM CaCl_2_, 0.8 mM MgSO_4_, and 5 mM glucose, buffered with 25 mM Hepes/Tris, pH 7.5) for 30 min. Cells were then washed with NaCl buffer and 200 µL of 100% ethanol was added to each well to lyse the cells and precipitate proteins. The plate was then kept in an incubator set at 37 °C to evaporate alcohol. The contents in the wells were then collected using 100 µL of nuclease-free water and spun down at 14,100 rpm for 30 min. The supernatant was collected and used for estimation of citrate using the BioVision citrate assay kit (San Francisco, CA, USA).

### Chromatin-immunoprecipitation (ChIP) assay

ChIP assays were performed using EZ-Magna ChIP A/G kit (Millipore, Burlington, MA, USA) according to the manufacturer’s instructions. Briefly, HepG2 cells were divided into three groups: no treatment, 10 mM metformin, and 1 mM AICAR. Following the treatment, cells were treated with 1% formaldehyde for 10 min to crosslink proteins and nucleic acids. The contents were then collected in 1X PBS supplemented with 1X Halt™ Protease and Phosphatase Inhibitor Cocktail (Thermo Fisher, Waltham, MA, USA) and lysed in nuclear lysis buffer. The lysate was then sonicated using BioRuptor Plus (Diagenode, Denville, NJ, USA) to shear DNA into fragments of approximately 200–1,000 base pairs. Chromatin concentration was measured using NanoDrop Spectrophotometer (Thermo Fisher Scientific) and 25 μg of DNA was used for immunoprecipitation with anti-SREBPF-1 antibody(Novus Biologicals LLC, Centennial, CO, USA), or normal mouse IgG (Millipore). Before immunoprecipitation, a small aliquot of the supernatant was removed as an input. DNA was isolated on the column and relative enrichment of SREBP-1 on *SLC13A5* promoter was assessed via PCR. The PCR primer sequences are provided in Supplementary Table S2.

### Western blot analysis

HepG2 cells were seeded at 3 ×10^6^ cells per 10-cm plate. Once the cells reached 75–80% confluency, HepG2 cells were divided into three groups: no treatment, 10 mM metformin, and 1 mM AICAR using physiological glucose levels (5 mM) for 24 h. Cells were collected in the 1X PBS supplemented with 1X Halt™ Protease and Phosphatase Inhibitor Cocktail (Thermo Fisher) and lysed in nuclear lysis buffer. The protein content of the solubilized nuclei was determined by Pierce™ BCA Protein Assay Kit (Thermo Fisher Scientific). Western blot samples were prepared in Laemmli sample buffer (Bio-Rad Laboratories). Protein (10 µg) was loaded onto a 10% SDS-polyacrylamide gel electrophoresis (SDS-PAGE) gel and transferred onto a PVDF membrane (Bio-Rad Laboratories). The membrane was blocked and antibodies were diluted in 5% non-fat dry milk (Bio-Rad Laboratories) or 5% bovine serum albumin (Irvine Scientific, Santa Ana, CA, USA). Anti-phospho-(Ser133)-CREB antibody (#17-10131) was purchased from Millipore-Sigma (St. Louis, MO, USA); Anti-Histone-3 antibody (#4499) and anti-phospho-(Ser2448)-mTOR antibody (# 2971S) were purchased from Cell Signaling Technology (Danvers, MA, USA). Horseradish peroxidase-conjugated goat anti-rabbit antibody (#1706515) was purchased from Bio-Rad Laboratories. Protein bands were visualized using Pierce™ ECL Western Blotting Substrate (Thermo Fisher Scientific) and developed on the autoradiography film (Santa Cruz, Dallas, TX, USA).

### Statistics

For experiments conducted in cell lines, measurements were made in triplicates, and the experiments were repeated 3–4 times. Data are expressed as mean values of independent experiments ± S.D. Statistical analyses and graphing were performed in GraphPad Prism 7.01 software (Graphpad, San Diego, CA, USA). Statistical differences between control groups and experimental groups were analyzed by one-way analysis of variance (ANOVA) followed by Dunnett’s test for multiple comparisons; in some experiments, a two-tailed, unpaired Student’s t-test for single comparison was used because of the single comparison and small sample size; the alpha- and p-value considered for significance was P < 0.05. For both statistical tests, the normality was confirmed using the GraphPad Prism 7.01 software (Graphpad).

## Supplementary information

Supplemental information.

## Data Availability

The data that support the findings of this study are available from the corresponding author (V.G.) upon reasonable request.
