# Supplementary material for: The Hepatic Plasma Membrane Citrate Transporter NaCT (SLC13A5) as a Molecular Target for Metformin
Source: Sci Rep. 2020 May 22;10:8536. doi: 10.1038/s41598-020-65621-w (PMC7244496; doi:10.1038/s41598-020-65621-w)

Supplementary Information

Title: The Hepatic Plasma Membrane Citrate Transporter NaCT (SLC13A5) as a Molecular Target for Metformin

Authors: Jonathan Kopel, Kei Higuchi, Bojana Ristic, Toshihiro Sato, Sabarish Ramachandran, and Vadivel Ganapathy

Author's Affiliation: Department of Cell Biology and Biochemistry, Texas Tech University Health Sciences Center, Lubbock 79430, Texas, United States

Supplementary Table 1. Effect of pretreatment of AICAR and metformin on kinetic parameters of citrate uptake in HepG2 cells

Pretreatment	LiCl	K_m (mM)	V_{max} (nmol/mg protein/30 min)
Control	(-)	6.39 ± 1.28	80.7 ± 10.0
1 mM AICAR	(-)	5.97 ± 1.58	39.4 ± 6.3
Control	(+)	0.482 ± 0.158	11.2 ± 1.4
1 mM AICAR	(+)	0.144 ± 0.034	4.53 ± 0.36
5 mM metformin	(+)	0.737 ± 0.437	3.85 ± 1.40

Each value represents the mean ± S.E.

Supplementary Table 2: Primers for chromatin Immunoprecipitation (ChIP)

Primer Name	Sequence
Human GAPDH	Fwd: 5’ -GTATTCCCCCAGGTTTACAT-3’<br>Rev: 5’ -TTCTGTCTTCCACTCACTCC-3’
Human LDL	Fwd: 5’ -CACTTTCGAAGGACTGGAGTGG-3’<br>Rev: 5’ -CCACGTCATTTACAGCATTTC-3’
Human SLC13A5 SREBP-1 Region I	Fwd: 5’ -TGCATCCCGGAGAAAAAGGT-3’<br>Rev: 5’ -TTCATCACCACCTGTCCAGC-3’
Human SLC13A5 SREBP-1 Region II	Fwd: 5’ -ACTTGTCTGAGGCACACAGC-3’<br>Rev: 5’ -TCCAAAGGGATTCACCAGAGC-3’

Supplementary Table 3: RT-PCR primers

Primer Name	Sequence
18S	Fwd: 5’ -CCCGTTGAACCCCATTCGT-3’<br>Rev: 5’ -GCCTCACTAAACCATCCAATCGGTA-3’
Human SLC13A5	Fwd: 5’ -CACCTTGTTCCTGCCCATCT-3’<br>Rev: 5’ -CCTGTTTTCACCATGTCAGCA-3’

Lack of additive effect of rapamycin when present together with metformin or AICAR during treatment in HepG2 cells

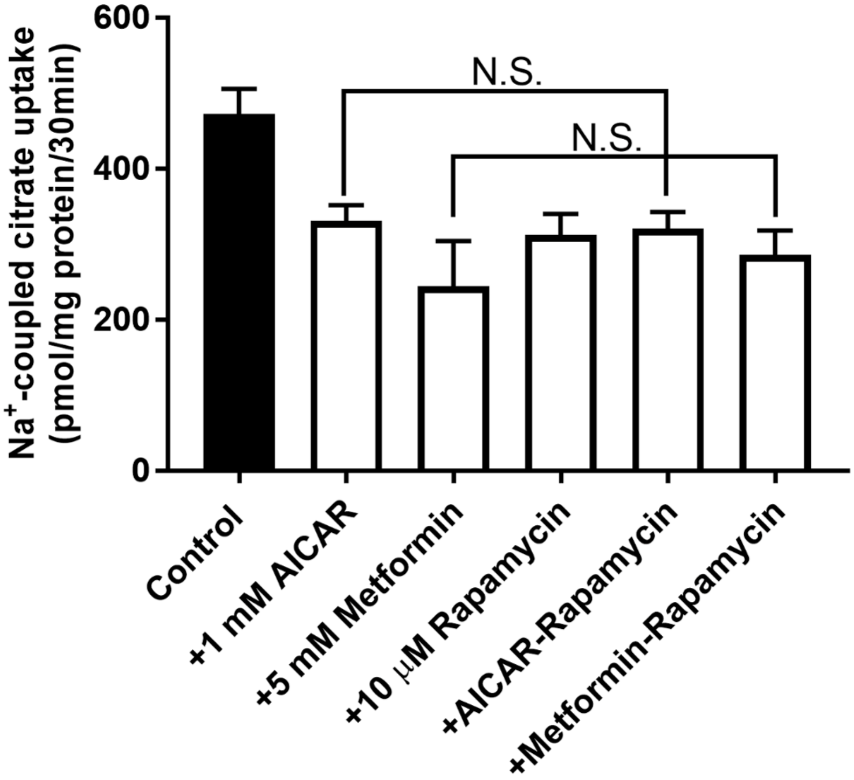

Supplementary Figure 1: HepG2 cells were cultured under conditions of physiologic concentration of glucose (5 mM), and treated with AICAR (1 mM), metformin (5 mM), rapamycin (10 µM), AICAR (1 mM) with rapamycin (10 µM) or metformin (5 mM) with rapamycin (10 µM) for 24 h. Uptake of [^{14}C]-citrate was measured in presence of 10 mM Li^+. Each column represents the mean ± S.D. (n = 12). In each case, citrate uptake in treated cells was significantly less than citrate uptake in untreated control cells ($P < 0.01$). N.S., not significant.

Chromatin Immunoprecipitation (ChIP) for SREBPF-1

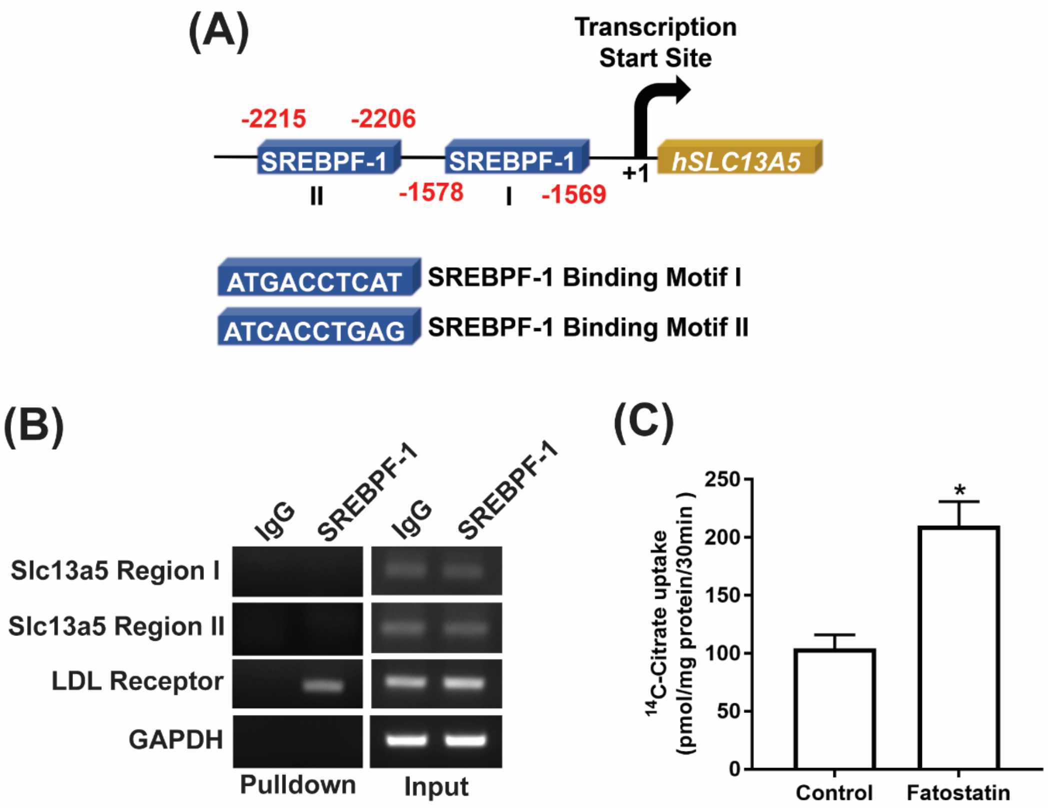

Supplementary Figure 2: Chromatin-Immunoprecipitation (ChIP) Analysis of SREBP-1. (A) Potential binding regions for SREBP-1 to SLC13A5 promoter. (B) ChIP results for SREBP-1. (C) [^{14}C]-Citrate uptake was measured in control and fatostatin-treated HepG2 cells. Cells were cultured in 20-mM glucose medium and treated with or without 40 µM fatostatin for 24 h prior to uptake measurement. Uptake was measured in transport buffer in presence of 10 mM Li^{+}. Each column represents the mean ± S.D. of nine determinations. *P<0.05 indicates a significant difference *vs* the control.

Whole agarose gel image of SREBPF-1 ChIP on *SLC13A5* and *LDL* promoters

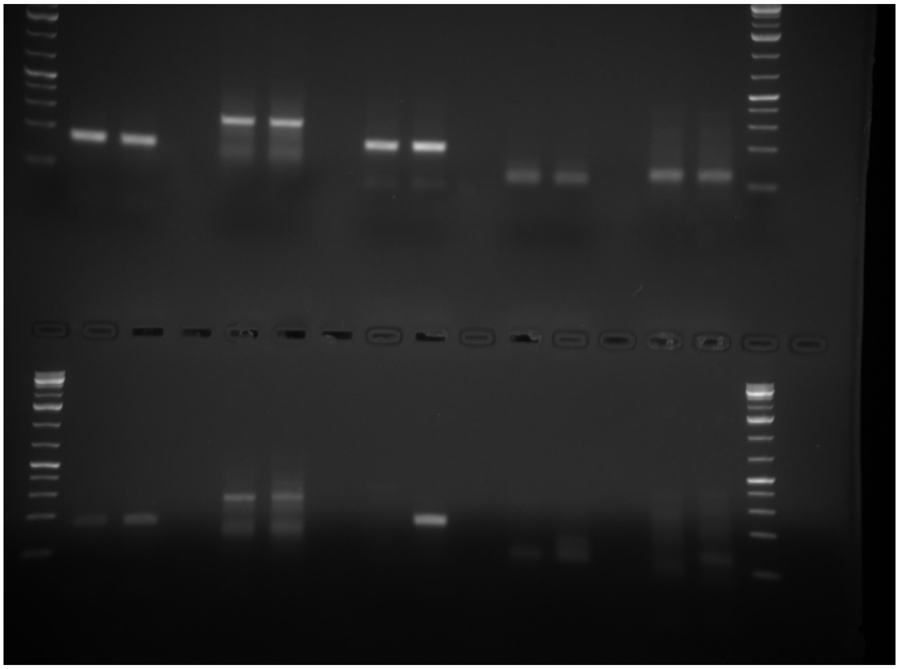

Supplementary Figure 3: Raw, full agarose gel of SREBPF-1 ChIP on *SLC13A5* and *LDL* promoters. Top row is the input. Lanes from the right: (**1-6**) Top and bottom are not used. Please disregard; (**7**) Promoter of *LDL* Receptor, IgG; (**8**) Promoter of *LDL* Receptor, SREBPF-1; (**10**) Promoter of *SLC13A5* Region I, IgG (**11**) Promoter of *SLC13A5* Region I, SREBPF-1; (**13**) Promoter of *SLC13A5* Region II, IgG (**14**) Promoter of *SLC13A5* Region II, SREBPF-1. The top row is the input, the bottom row is the pulldown. Lanes (**3, 6,9,12**) are empty. Cropped image is used in the Supplementary Figure 2.

Whole agarose gel image of SREBPF-1 ChIP on *GAPDH* promoter

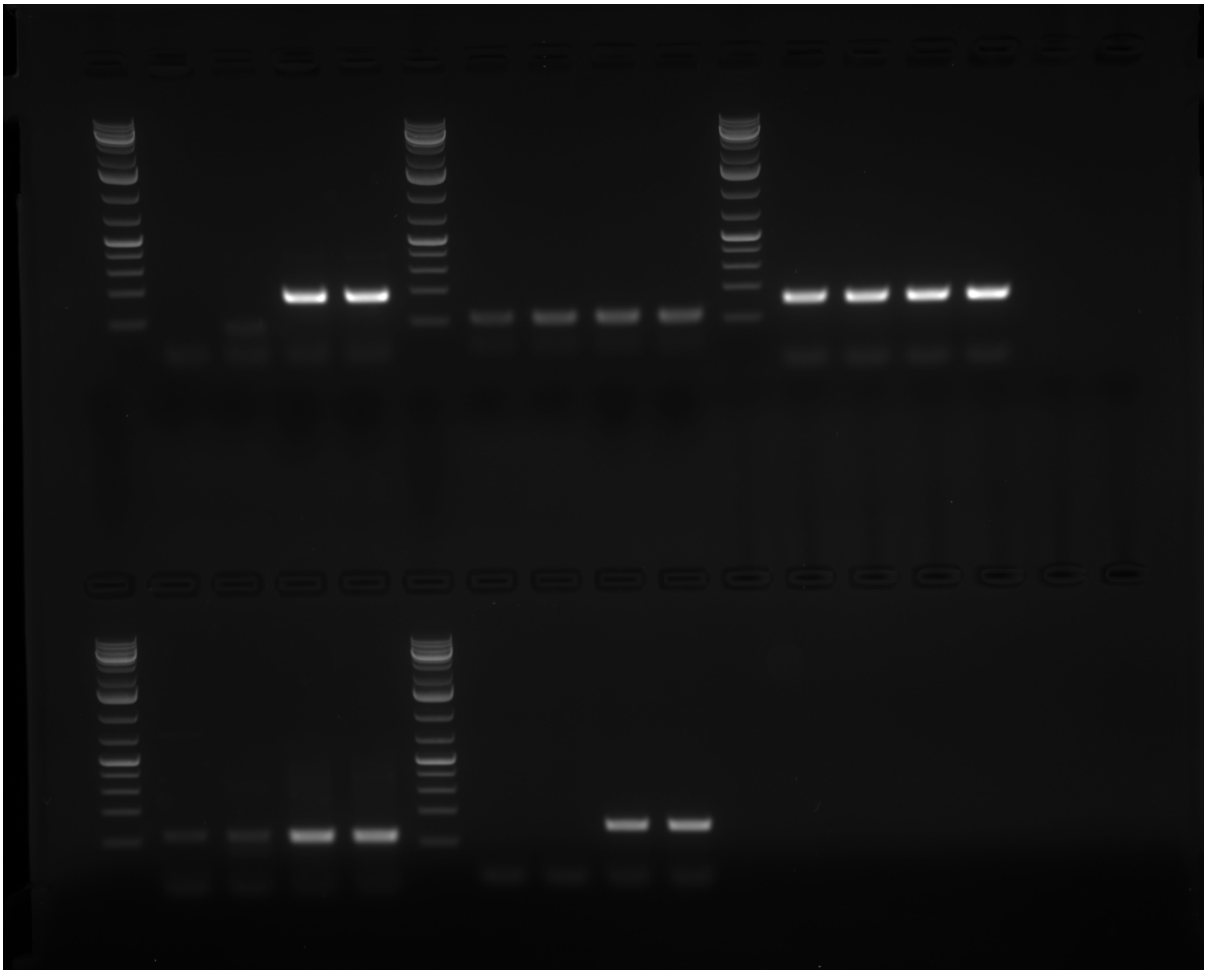

Supplementary Figure 4: Raw, full agarose gel of SREBPF-1 ChIP on *GAPDH* promoter. Only top row, lanes (**1-4**) are used. Please disregard the rest. Top row lanes: (**1**) Promoter of *GAPDH*, IgG pulldown (**2**) Promoter of *GAPDH,* SREBPF-1 pulldown; (**3**) Promoter of *GAPDH*, IgG input; (**4**) Promoter of *GAPDH*, SREBPF-1 input. Cropped image is used in the Supplementary Figure 2.

Whole phospho-mTOR western blot

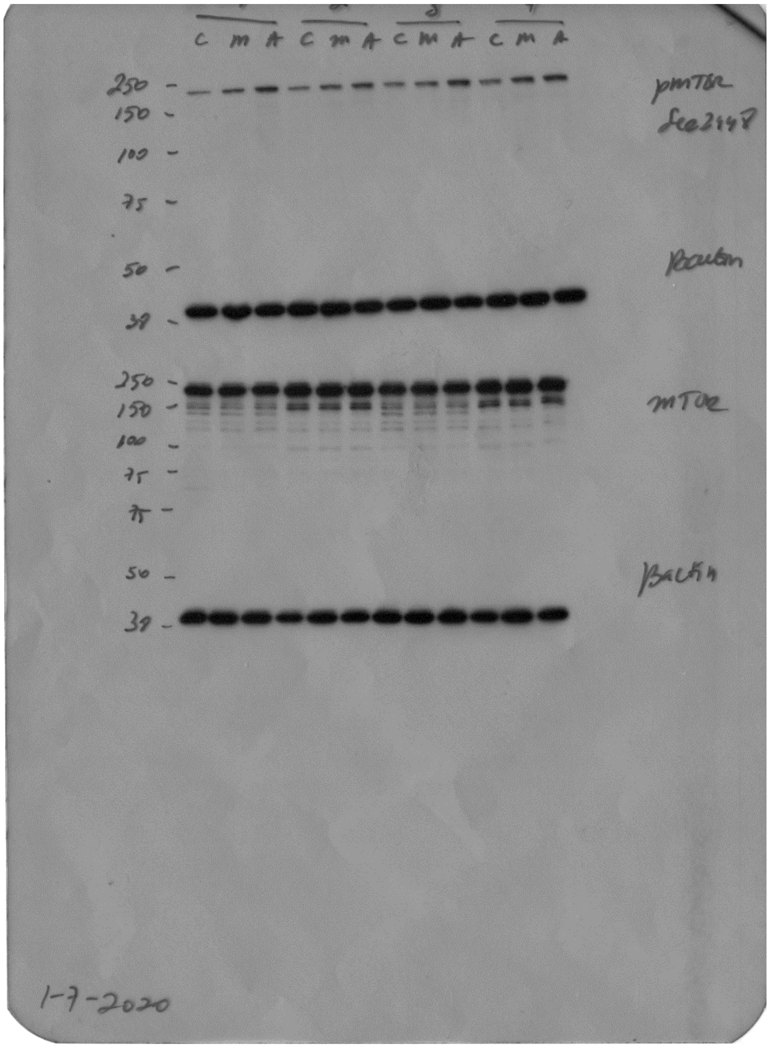

Supplementary Figure 5: Raw, full Western Blot for phospho-mTOR (p-mTOR)**.** Rows from the top: (**1**) p-mTOR in control (lanes 1,4,7,10), 1 mM Metformin (lanes 2, 5, 8, 11) and 10 mM AICAR (lanes 3, 6, 9, 12). The experiment was performed in quadruplicates. p-mTOR is a 289 kDa protein. Cropped figure is used in Figure 6; (**2**) β-actin in control (lanes 1, 4, 7, 10), 1 mM Metformin (lanes 2, 5, 8, 11) and 10 mM AICAR (lanes 3, 6, 9, 12). The experiment was performed in quadruplicates. β-actin is a 42 kDa protein. Not used due to high exposure (**3**) Total mTOR in control (lanes 1, 4, 7, 10), 1 mM Metformin (lanes 2, 5, 8, 11) and 10 mM AICAR (lanes 3, 6, 9, 12). The experiment was performed in quadruplicates. mTOR is a 289kDa protein. Not used due to high exposure; (**4**) Not used. Please disregard.

Whole mTOR and β-actin western blot

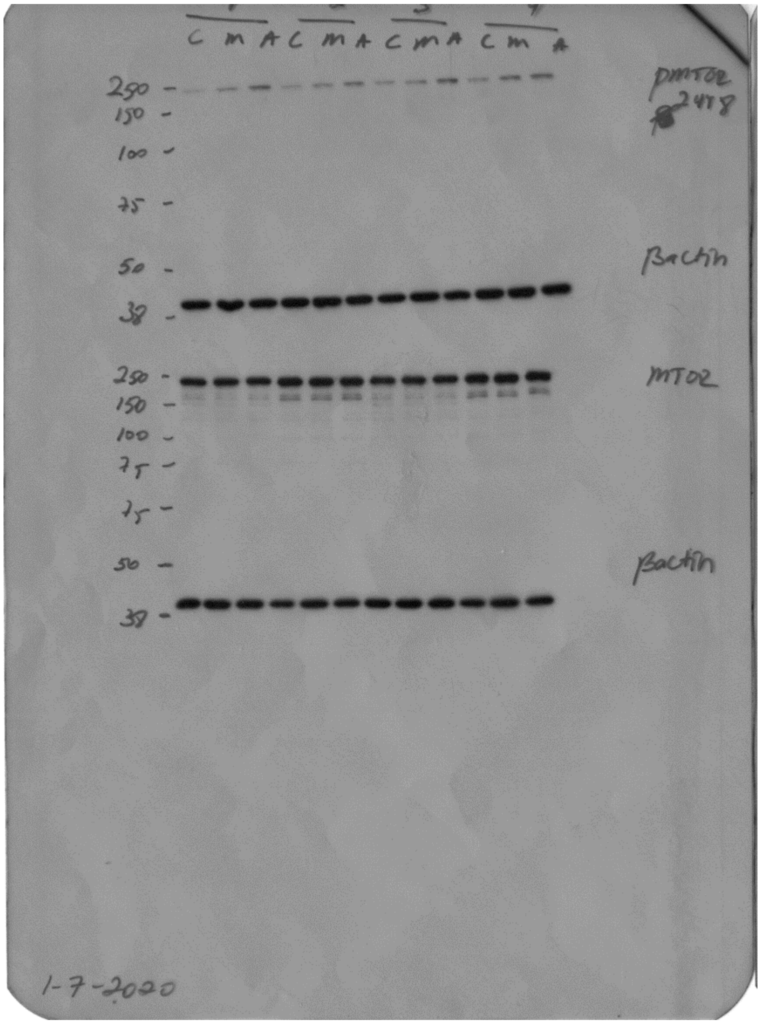

Supplementary Figure 6: Raw, full Western Blot for mTOR and β-actin. Rows from the top: (**1**) p-mTOR in control (lanes 1, 4, 7, 10), 1 mM Metformin (lanes 2, 5, 8, 11) and 10 mM AICAR (lanes 3, 6, 9, 12). The experiment was performed in quadruplicates. p-mTOR is a 289 kDa protein. Not used due to low exposure; (**2**) β-actin in control (lanes 1, 4, 7, 10), 1 mM Metformin (lanes 2, 5, 8, 11) and 10 mM AICAR (lanes 3, 6, 9, 12). The experiment was performed in quadruplicates. β-actin is a 42 kDa protein. Cropped figure is used in Figure 6; (**3**) Total mTOR in control (lanes 1, 4, 7, 10), 1 mM Metformin (lanes 2, 5, 8, 11) and 10 mM AICAR (lanes 3, 6, 9, 12). The experiment was performed in quadruplicates. mTOR is a 289 kDa protein. Cropped figure is used in Figure 6; (**4**) Not used. Please disregard.

Whole phospho-CREBP western blot

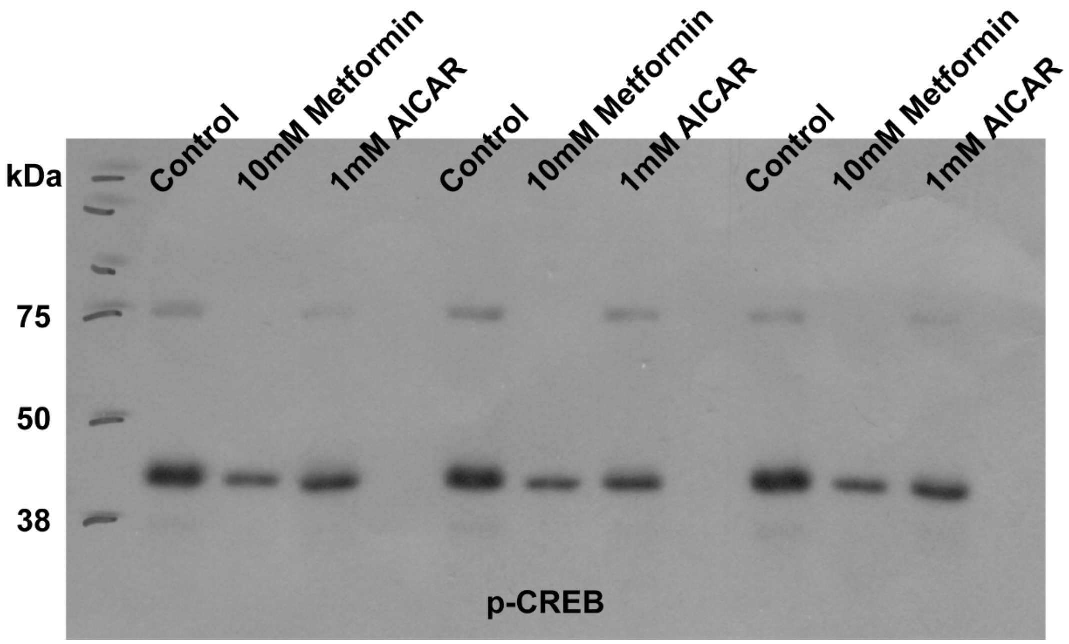

Supplemental Figure 7: Phospho-CREBP is a 43 kDa protein. Cropped figure is used in Figure 7.

Whole histone-3 western blot

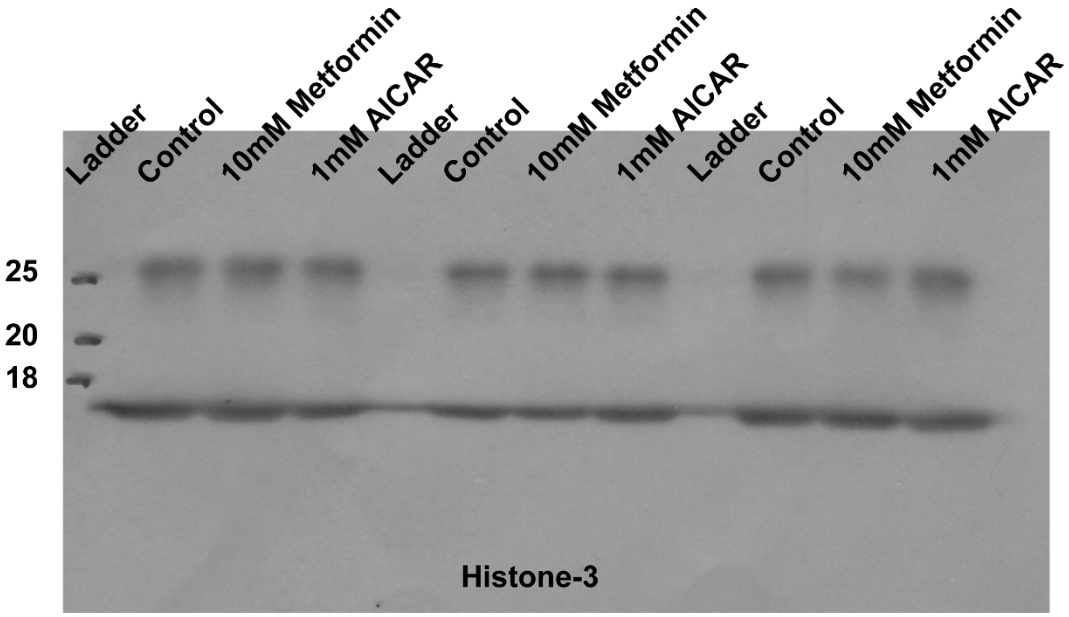

Supplemental Figure 8: Histone-3 is a 17 kDa protein. Cropped figure is used in Figure 7.

Supplementary Figure 9: Whole gel images for RT-qPCR

(a) 20 mM Glucose; Control, AICAR, and Metformin

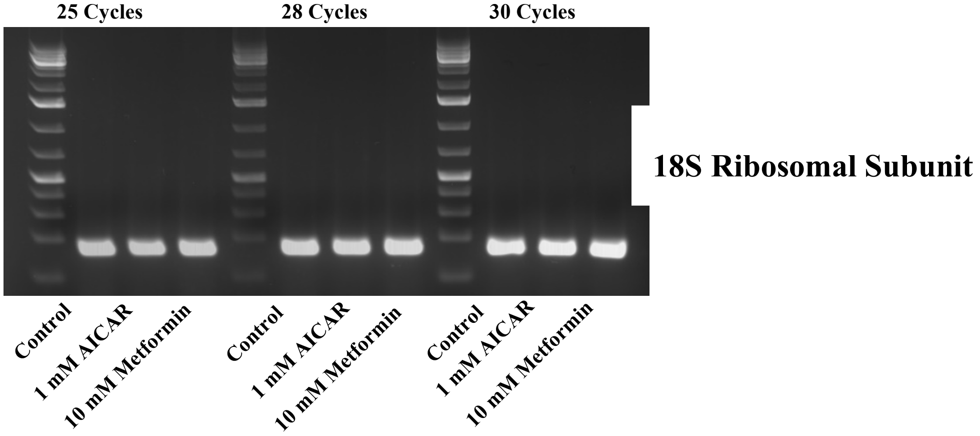

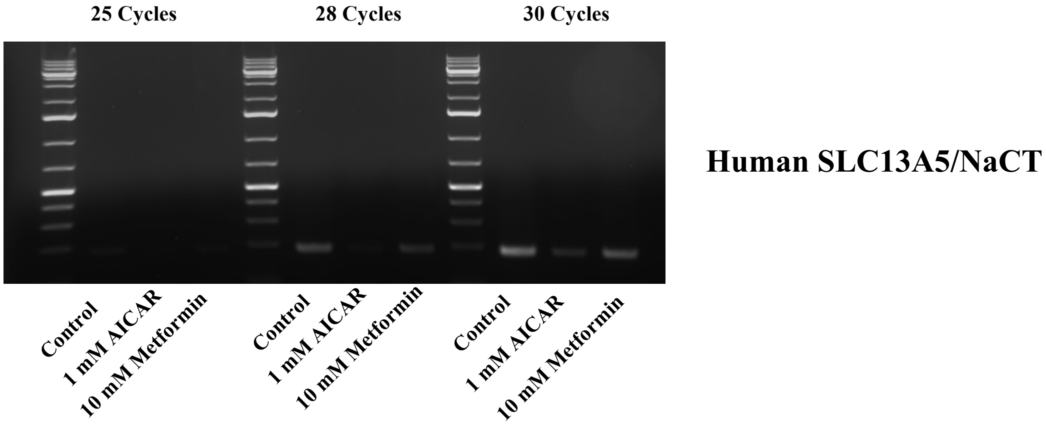

(b) 5 mM Glucose; Control, AICAR, and Metformin

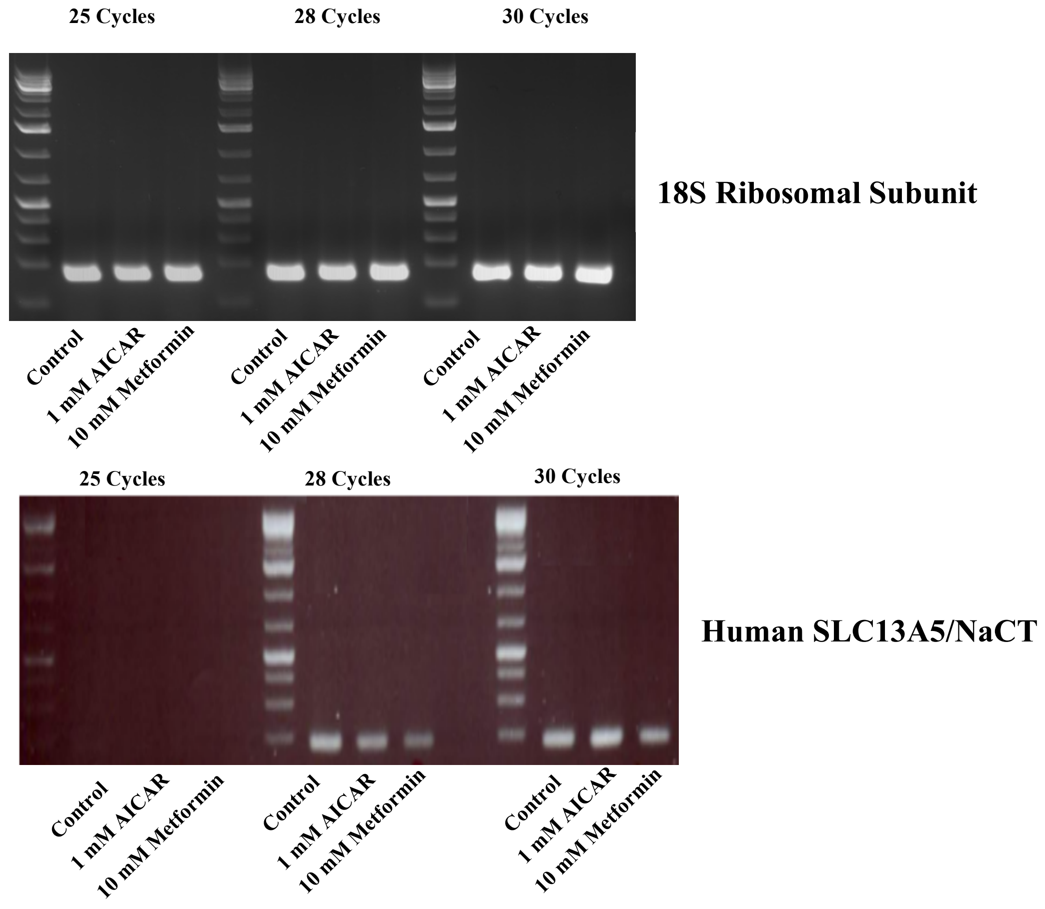

(c) 20 mM Glucose; Rapamycin

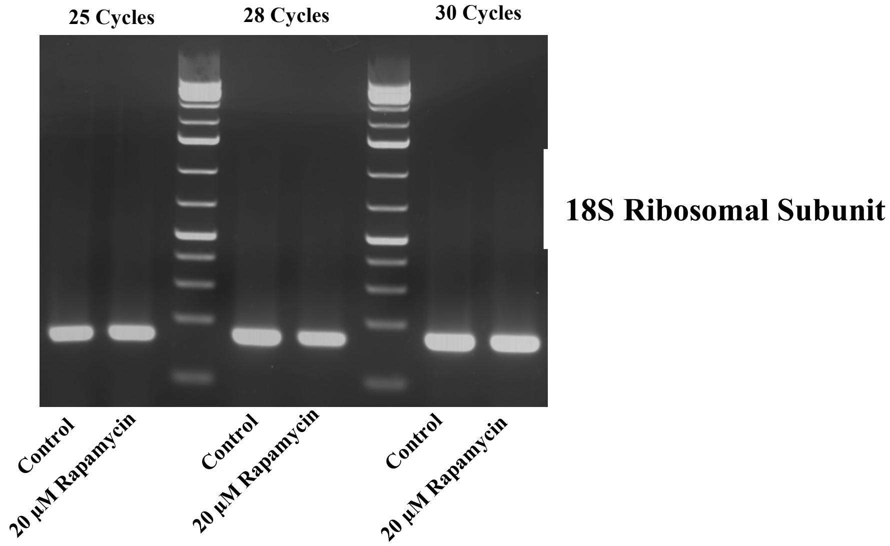

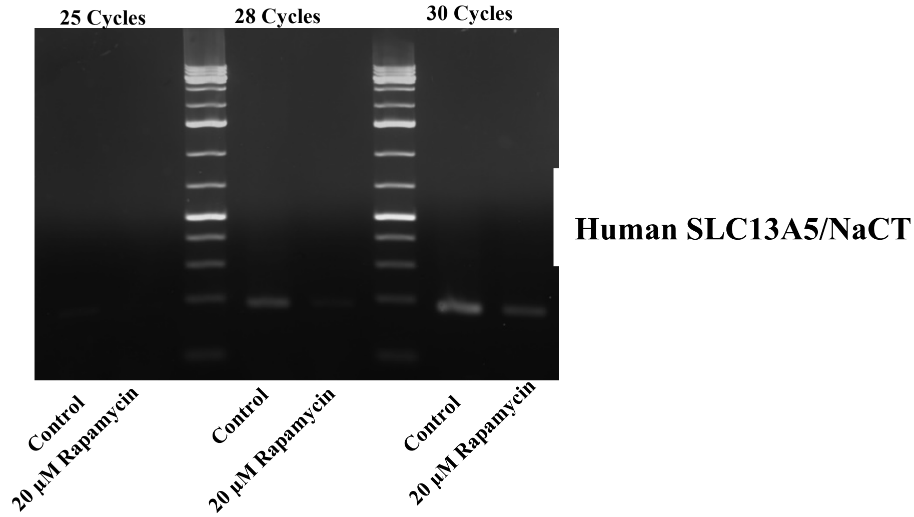

(d) 5 mM Glucose; Rapamycin

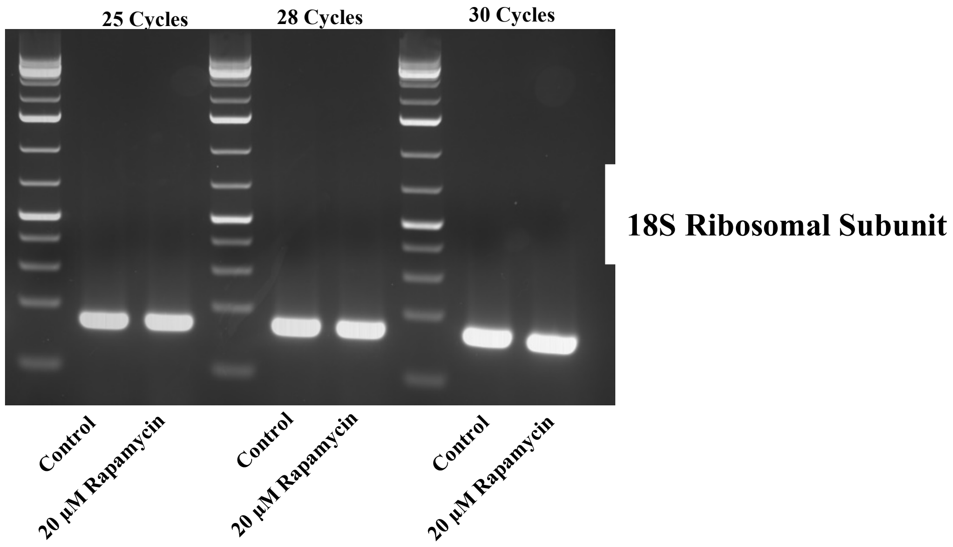

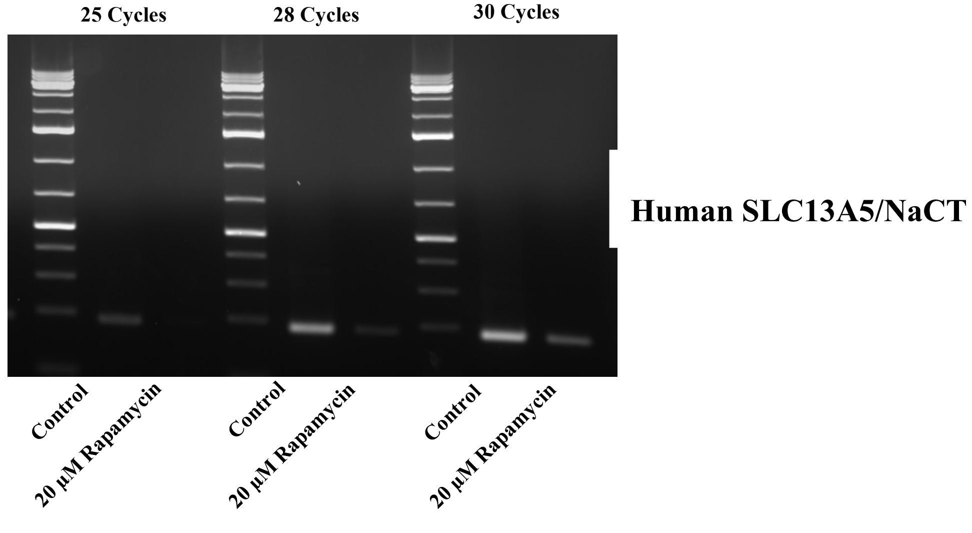

Supplement: Supplementary file 1 — Supplemental information. [file 41598_2020_65621_MOESM1_ESM.pdf]
